# Genotypic Characterization of Emerging Avian Reovirus Genetic Variants in California

**DOI:** 10.1038/s41598-019-45494-4

**Published:** 2019-06-27

**Authors:** S. Egaña-Labrin, R. Hauck, A. Figueroa, S. Stoute, H. L. Shivaprasad, M. Crispo, C. Corsiglia, H. Zhou, C. Kern, B. Crossley, R. A. Gallardo

**Affiliations:** 1 University of California, Davis, School of Veterinary Medicine, Davis, 95616 CA United States; 2 Auburn University Department of Pathobiology and Department of Poultry Science, Auburn, 36832 AL USA; 3 University of California, Davis, California Animal Health & Food Safety Laboratory System, 95380 CA Turlock, USA; 4 University of California, Davis, California Animal Health & Food Safety Laboratory System, 93274 CA Tulare, USA; 5 University of California, Davis, California Animal Health & Food Safety Laboratory System, 95616 CA Davis, USA; 6 Foster Farms, Livingston, 95334 CA USA; 7 University of California, Davis, School of Agriculture, Davis, 95616 CA United States

**Keywords:** Viral epidemiology, Nutrition disorders

## Abstract

This study focuses on virus isolation of avian reoviruses from a tenosynovitis outbreak between September 2015 and June 2018, the molecular characterization of selected isolates based on partial S1 gene sequences, and the full genome characterization of seven isolates. A total of 265 reoviruses were detected and isolated, 83.3% from tendons and joints, 12.3% from the heart and 3.7% from intestines. Eighty five out of the 150 (56.6%) selected viruses for sequencing and characterization were successfully detected, amplified and sequenced. The characterized reoviruses grouped in six distinct genotypic clusters (GC1 to GC6). The most represented clusters were GC1 (51.8%) and GC6 (24.7%), followed by GC2 (12.9%) and GC4 (7.2%), and less frequent GC5 (2.4%) and GC3 (1.2%). A shift on cluster representation throughout time occurred. A reduction of GC1 and an increase of GC6 classified strains was noticed. The highest homologies to S1133 reovirus strain were detected in GC1 (~77%) while GC2 to GC6 homologies ranged between 58.5 and 54.1%. Over time these homologies have been maintained. Seven selected isolates were full genome sequenced. Results indicated that the L3, S1 and M2 genes, coding for proteins located in the virus capsid accounted for most of the variability of these viruses. The information generated in the present study helps the understanding of the epidemiology of reoviruses in California. In addition, provides insights on how other genes that are not commonly studied add variability to the reovirus genome.

## Introduction

Avian reoviruses (ARV) are non-enveloped and possess a double-stranded, segmented ribonucleic acid (RNA) genome. They are members of the family *Reoviridae*, subfamily *Spinareovirinae* and genus *Orthoreovirus*. The ten different segments that have been identified and classified based on their electrophoretic mobility are: L1-L3, M1-M3 and S1-S4. The S1 segment encodes for three viral proteins, including the minor capsid protein σC^1^. This particular protein plays a key role during early stages of infection, mediating the interaction between the virion and the host cell, and elicits type-specific neutralizing antibodies^2,3^. Amplification and sequencing analysis of the portion of the S1 gene that encodes the σC protein, has been commonly used as a genetic marker for the characterization and classification of ARV isolates^4–7^. To date, five and six genotypes have been described based on Lu and Kant’s classification, respectively^2,6^.

Extreme variability is an inherent characteristic of ARV. This is based on their RNA nature and their segmented genome favouring mutations, recombination and reassortment events^8,9^. Since 2011, the poultry industry worldwide has been facing the consequences of the emergence of ARV variants^6,7,10–12^. These variants of ARV have been linked to severe viral arthritis, tenosynovitis and pericarditis mainly in vaccinated broiler chickens and their breeders^13,14^. The above mentioned pathological outcomes, in addition to subclinical presentations of the disease^10^ cause severe economic losses to the poultry industry. The affected productive parameters on ARV diseases are represented by reduced weight gain, lack of flock uniformity, impaired feed conversion rates, increased condemnations in the processing plants and welfare issues related to lameness^15^. In California, the reovirus tenosinovitis outbreak started in August 2015. It has affected broilers ranging from 14 to 47 days of age. Clinically, broiler flocks reported lameness due to deviation of legs either laterally or anteriorly, stunting and lack of uniformity. Most of the broilers had swollen hock joints with increased exudate that extended along the tendon sheath. Morbidity ranged between 0.3 to 15% and mortality ranged from 0.1 to 1%.

Despite the better understanding of the biology of the virus, their variability and the efforts of several groups across the U.S.^5,6,16,17^, Europe^14,18^, Canada^19,20^ and China^10^ in detecting and typing ARV variants, classical vaccine strains used for immunization of commercial flocks, namely S1133, 1733 and 2408, have not changed since the 1970’s. These strains have proven to be inefficient in controlling the infection, partly due to the RNA virus nature being prone to mutation and recombination events and generating variants that are partially or incompletely protected by antibodies generated by classical vaccine strains. The generation of variant strains that circumvent vaccine immunity, perpetuate the cycle of variability, and enhances the need for prompt detection, typing and autogenous vaccine formulation^5,18,21^. The first step to generate control and prevention strategies against reoviruses is to be able to characterize the strains causing disease in the field and based on that characterization select virus strains to be included in autogenous vaccines.

This study focused on virus isolation of avian reoviruses between September 2015 and June 2018, the molecular characterization of selected reoviruses based on partial S1 gene sequences, and the full genome characterization of seven selected isolates. These detection and characterization efforts have generated a molecular surveillance system that can be used to assess the variability of reoviruses in the field and guide virus selection for vaccine production in the State of California.

## Results

### Virus isolation

From September 2015 to June 2018, we received 265 commercial broiler cases where ARV was detected by Reverse transcriptase polymerase chain reaction (RT-PCR) followed by virus isolation and tenosynovitis was confirmed by histopathological findings. ARV isolates were obtained from tendons (78.9%), heart (12.3%), joints (4.4%), intestines (3.7%), liver (0.35%) and pancreas (0.35%).

### Molecular characterization

**Figure 1:**
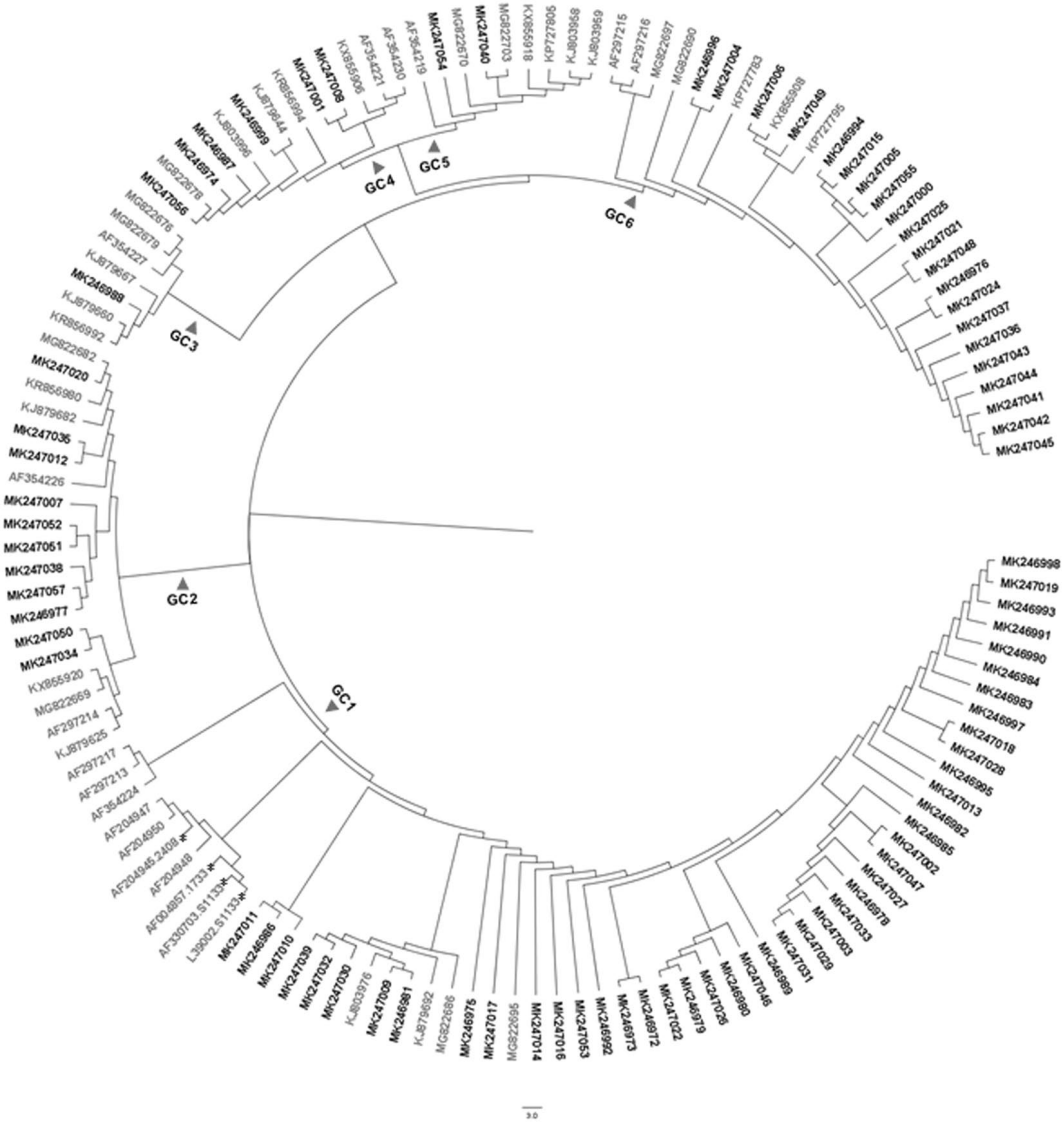
Phylogenetic tree depicting 85 ARV S1 sequences (278 AA). Sequences were obtained from reoviruses isolated from tenosynovitis cases in CA between 2015 and 2018. The reference sequences (gray) were obtained from GenBank. Outbreak sequences (Black) were grouped into six genotypic clusters (GC). Commercial vaccine strains were labeled by asterisks.

**Figure 2:**
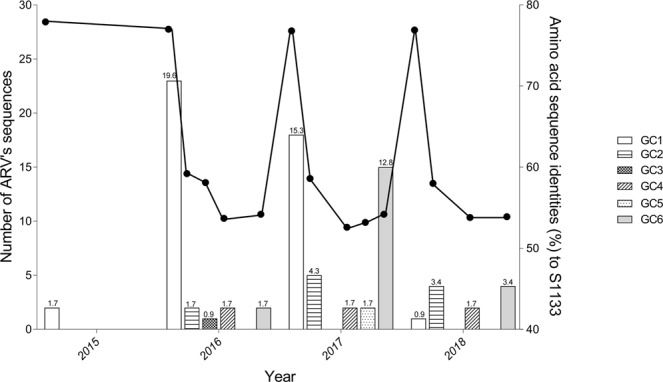
Frequencies and average homologies based on 85 ARV S1 sequences (278 AA) obtained from avian reovirus (ARV) isolates from tenosynovitis clinical cases. Bars are showing the isolate frequencies in each genotypic cluster (GC) per year. Numbers above bars represent the percentage (%) from the total samples. The bold line represents the average homology to S1133 based on the S1 sequences. GC 1 to 6 represents genotypic clusters 1 to 6.

One hundred and fifty isolates were selected for molecular characterization. The selection criteria involved clinical relevance, gross pathology and cytopathic effect in chicken embryo liver cells (CEL). Effective amplification of a 1,088 bp segment of the ARV S1 gene was accomplished in 85 out of the 150 selected reovirus isolates (56.6%) using RT-PCR. All partial S1 sequences were uploaded to GenBank, accession numbers are located in Supplementary Table 1. The obtained sequences were aligned and subsequently used to construct a phylogenetic tree, including 49 reference sequences representing the genotypic groups, 1 to 6, obtained from the available literature^2,6,9,11,20,22^ (Fig. 1). GenBank accession numbers for the sequences added as reference in the phylogenetic tree can be found in Supplementary Table 2. The typed viruses grouped in six different genotypic clusters (GC); two of these, GC1 and GC6, were predominant. In percentage, the distribution of the sequences by GC was as follows: GC1 (51.8%), GC2 (12.9%), GC3 (1.2%), GC4 (7.1%), GC5 (2.4%) and GC6 (24.7%) (Table 1). The percent amino acid homology of the S1 sequences to the reference ARV S1133 strain were: GC1 (77.0%), GC2 (58.5%), GC3 (58.0%), GC4 (53.5%), GC5 (53.1%) and GC6 (54.1) (Table 2). The percent homologies of the S1 sequences to S1133 by year from 2015 to 2018 showed consistency; GC1 was the group with the highest homology to S1133 (77.0%) while GC4, GC5 and GC6 the groups with the lowest homologies (53.1 to 54.1%). In order to assess the similarity of the S1 sequences within each cluster we calculated the average of the pairwise homologies between all S1 gene sequences in a cluster i.e. homology within GC. From high to low, these homologies were GC5 (97.8%), GC1 (96.4%), GC6 (94.8%), GC4 (77.3%) and GC2 (76.4%) (Table 2). A summary of the sequence distribution on the different genetic clusters and their homologies by year were summarized in Fig. 2. A considerable reduction of sequences clustered in GC1 from 76.7% in 2016 to 9.1% in 2018, was followed by an increase in GC2, GC4 and GC6 from 6.7% in 2016 to 36.4%, 36.4% and 18.2%, respectively in 2018. GC3 was first identified in 2016 with 3.3% of the sequences disappearing in 2017. Finally, GC5 was first identified in 2017 with 4.8% of the sequences disappearing in 2018.

**Table 1:** Sequence frequencies by genotypic cluster (GC) and year from 2015 to 2018, arithmetic sum and percentage of the total sequences by genotypic cluster (ND = non-detected).

Genotypic cluster	Total sequences by year				Sum	Total (%)
	2015	2016	2017	2018		
GC1	2	23	18	1	44	51.8
GC2	ND	2	5	4	11	12.9
GC3	ND	1	ND	ND	1	1.2
GC4	ND	2	2	2	6	7.1
GC5	ND	ND	2	ND	2	2.4
GC6	ND	2	15	4	21	24.7

ND = not detected.

**Table 2:** Amino acid identities (%) between S1133 and the 85 ARV isolates based on sigma C protein by year (ND = non-detected).

Genotypic cluster	AA identity (%)				Average (%)	AA identity within GC (%)
	2015	2016	2017	2018		
GC1	77.9	77.0	76.8	76.9	77.0	96.4
GC2	ND	59.2	58.6	58.0	58.5	76.4
GC3	ND	58.0	ND	ND	58.0	ND
GC4	ND	53.7	52.6	53.8	53.5	77.3
GC5	ND	ND	53.1	ND	53.1	97.8
GC6	ND	54.2	54.2	53.9	54.1	94.8

ND = not detected.

### Whole genomes

**Figure 3:**
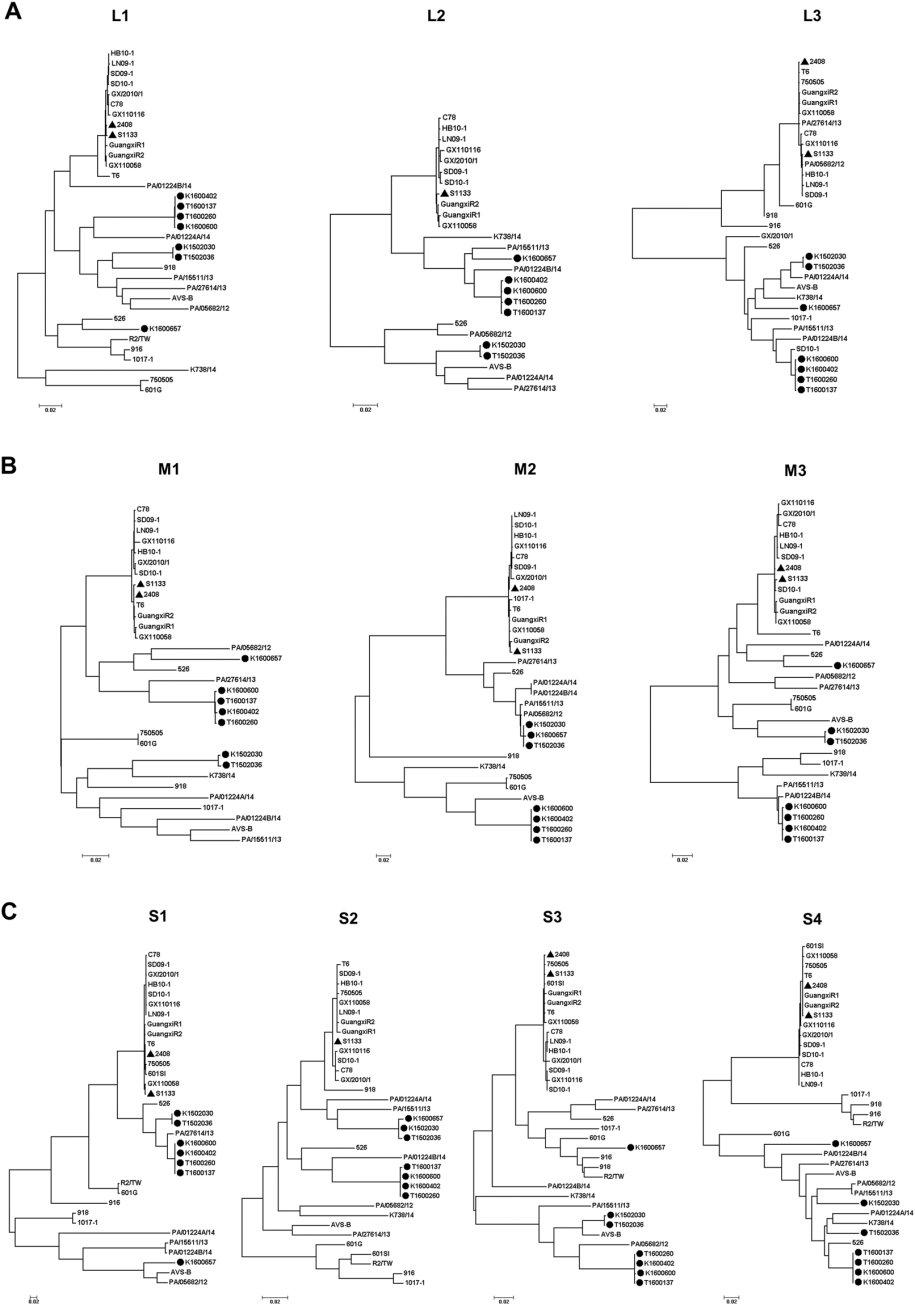
Phylogenetic trees showing each gene of seven California ARV’s isolates. Twenty-eight sequences were obtained from GenBank and used as reference strains. Amino acid sequences were aligned and trimmed: L1 (1,291 AA); L2 (1,260 AA); L3 (1,218 AA); M1 (677 AA); M2 (677 AA); M3 (636 AA); S1 (514 AA); S2 (280 AA); S3 (367 AA); S4 (366 AA) phylogeny was performed using the Maximum Likelihood Method with 1,000 bootstrap replicates using MEGA 7. The commercial vaccine strain (S1133 and 2408) are labelled with a triangle. Black dots are showing the clustering of the California isolates.

Seven ARV isolates K1600600, K1600402, K1502030, K1600657, T1502036, T1600137 and T1600260, associated with severe clinical signs in the field, were selected for full genome studies. These viruses molecularly grouped into GC1, except K1600657 that grouped into GC4 under a partial S1 gene molecular characterization. The goal was to determine how variable were the genes that are not frequently used for avian reovirus molecular characterizations and how much each gene contributes to the reovirus variability. Information about the raw reads after NGS, non-host reads, contigs, viral contigs, viral reads and % viral reads are summarized in Table 3. The percent identity of each of the genes of these viruses to ARV S1133 genes was calculated (Table 4). Considering average gene identities to S1133 among the seven sequences, the lowest detected identities were found in: L3 gene, coding for γC an inner and outer capsid protein (72.7%); S1 gene, coding for σC a minor capsid protein (77.9%); and finally M2 coding for μB an outer-capsid protein (79.0%). Additionally, phylogenetic trees were made to graphically compare the differences and clustering of the ARV’s by gene. Figure 3 shows the trees prepared with all gene segments corresponding to the ARV genes analysed. The California strains in all gene trees grouped distantly to the vaccine cluster defined by KF741758 (S1133) (Fig. 3). California molecular variants T1600137, T1600260, K1600402 and K1600600 formed a sub-cluster. Two of the seven California ARV’s: K1502030 and T1502036 form a different cluster, while K1600657 doesn’t cluster with any of the CA viruses. The clustering pattern apply to all phylogenetic trees constructed except in M2, S2 and S4. The M2 gene AA sequences grouped in two defined clusters, one containing K1502030, K1600657 and T1502036 while the other contained K1600600, K1600402, T1600260 and T1600137. In S2 two distinct clusters were formed one containing K1502030, K1600657 and T1502036 and the other T1600137, T1600260, K1600402 and K1600600. In the case of S4, K1502030 and T1502036 clustered in different sub-clusters but close together in the phylogenetic tree.

**Table 3:** Raw reads, non-host reads, contigs, viral contigs, viral reads and % viral reads obtained after processing the information obtained after the NGS.

Strain	Raw reads	Non-host reads	Contigs	Viral contigs	Viral reads	% viral reads
K1600600	17931054	446486	816	11	2578	0.01438%
K1600402	23077166	553129	802	11	5404	0.02342%
K1502030	21005141	557814	686	11	3712	0.01767%
K1600657	23693462	567956	958	11	3760	0.01587%
T1502036	21193861	534662	1008	11	4953	0.02337%
T1600137	22793555	537209	602	11	33228	0.14578%
T1600260	23297817	544087	1218	11	5933	0.02547%

**Table 4:** Amino acid sequence identities (%) between S1133 and each of the genes of seven whole genome sequences from selected ARV isolates.

Isolate ID	Viral segment	L 1	L 2	L 3	M1	M2	M3	S1	S2	S3	S4
	Encoded proteins	λA^a^	λB^a^	λC^b^	μA^a^	μB^c^ μBN^c^ μBC^c^	μNS^d^ μNSC^d^ μNSN^d^	σC^c^ p10^d^ p17^d^	σA^a^	σB^c^	σNS^d^
K1600600		88.1	90.1	73.0	89.3	74.7	81.2	81.5	89.9	85.1	81.4
K1600402		88.1	90.1	73.0	89.3	74.8	81.1	81.3	89.9	85.2	81.4
K1502030		88.7	83.8	72.2	87.2	84.7	87.4	80.8	91.6	84.9	80.0
K1600657		89.2	89.4	72.9	88.2	84.5	89.3	58.4	91.2	88.5	79.6
T1502036		88.7	83.7	72.2	87.1	84.7	87.5	80.7	91.3	85.0	81.4
T1600137		88.1	90.1	73.0	89.4	74.7	81.3	81.4	89.9	85.1	81.4
T1600260		88.1	90.1	72.9	89.4	74.7	81.2	81.3	89.9	85.2	81.4
	**Average identity (%)**	**88**.**4**	**88**.**2**	**72**.**7**	**88**.**6**	**79**.**0**	**84**.**2**	**77**.**9**	**90**.**5**	**85**.**6**	**81**.**0**

Superscripts indicate: a = Inner core, b = Inner capsid and outer capsid, c = Outer capsid, d = Non-structural.

## Discussion

Molecular surveillance is crucial to strategize control and prevention of endemic diseases. This is particularly important for reoviruses considering their segmented RNA genome and their potential for variation^2,6,7,11,12^. Since 2015, the isolation of reoviruses causing tenosynovitis in broiler chickens obtained from breeders vaccinated with live and inactivated conventional strains (S1133, 1733, 2408, etc.) has raised the concern of the existence of reovirus genetic variants in California.

The first step in surveillance is pathogen detection and isolation. Since diagnostic RT-PCR’s focuses on a conserved segment of the ARV genome i.e. S4^23^ will not be able to differentiate variant strains. This is when virus culture/isolation and subsequent virus genomic characterization, from highly variable genes, becomes crucial in the surveillance strategy. In this project, in a period of 3 years, we were able to detect and isolate 265 reoviruses. The highest percentage of recovery was from tendons and joint tissues or swabs (83.3%) followed by heart (12.3%) and intestines (3.7%), liver and pancreas provided lower virus recovery. Other studies have focused on tendons and joint tissues for their typing work, without reporting the isolation effectiveness in these tissues^6,20^.

The reovirus isolation method involves passages on CEL cells. Passaging RNA viruses in cells involve genetic changes as part of the adaptation of these viruses to the a new cell culture or host^24^. We assume that those changes in the studied reoviruses are minimum, since only one and at the most two passages in cells were performed for the obtention of cytopathic effect (CPE) and the subsequent virus isolation.

Sequencing analysis of a portion of the S1 gene has been commonly used as a genetic marker for the characterization and classification of ARV isolates^4^. From the isolated reovirus strains, 150 were selected for partial S1 genotypic characterization based on clinical relevance, gross pathology and cytopathic effect in chicken embryo liver cells (CEL). Using the primers described by Kant *et al*.^2^, only 85 out of 150 (56.6%) partial S1 genes were detected, amplified and sequenced. We attribute the lack of amplification of more than 30% of the isolates partially to the molecular divergence on ARV variant strains. Kant *et al*., using the same primers and viral isolates between 1980 to 2000, had a higher amplification success i.e. (28/40) 70%^2^.

Partial S1 gene characterization methods have classified ARV strains into five^2,11^ and/or six^6,12,20^ genotypic clusters. We performed a phylogenetic analysis based on deduced amino acid sequences to take into account synonymous and non-synonymous mutations. Our results showed that reovirus strains isolated in California belong to all six distinct genotypic clusters (GC1 to GC6). These clusters were clearly defined and confirmed by the addition of reference sequences representing earlier ARV isolates and vaccine strains. The most predominant clusters were GC1 (51.8%) and GC6 (24.7%), followed by GC2 (12.9%), GC4 (7.1%) and with lower frequencies GC5 (2.4%) and GC3 (1.2%). Similar results were described in Europe by Kant. Most of the isolates associated with malabsorption syndrome belonged to clusters 1 and 4 and few in clusters 2 and 5. Most the tenosynovitis clusters belonged to cluster 4 and unclear cases to cluster 1^2^. Different molecular characterization results were reported by Lu *et al*.^6^ and Palomino *et al*.^20^. While Lu stated that most of their sequences clustered in GC2, followed by GC4 and GC1, Palomino affirmed that their most predominant sequences were from GC5, followed by GC4 and GC1. While this “cluster” nomenclature is used to compare the viruses detected in different parts of the country and the world, we need to take into consideration that fragment size, the number of sequences in the analysis, sequences selected as reference strains and the subjectivity of the analysis play a role in the formation of the clusters. Genetic variants detected in California follow the same S1 gene phylogenetic classification than the S1 sequences used as references. Some interesting points to consider are that older isolates from Taiwan, i.e. 1970, reported by Liu’s group^9^, cluster closer to vaccine strains S1133 and 1733. Isolates after 1986, group throughout the 6 genetic clusters and far from the subgroup of GC1 containing the vaccine strains. Interestingly, two Taiwanese strains from 1992 reported by Liu^9^ grouped in GC6, these are the oldest strains classified in this distant group. These results might be suggesting major genetic changes occurred starting in 1986 generating genetic variants from the “conventional vaccine types” of reovirus.

Between 2015 and 2018, the ARV isolates genotypic cluster representation in the State of California has shifted. A decrease on the representation of GC1 and an increase of GC6 classified strains has occurred (Table 1). Multiple factors might be influencing this relevant shift, including the use of autogenous vaccines. The use of certain GC’s as antigen in autogenous vaccines might be important in driving the change in the representation of the different ARV genetic clusters causing disease in the field. While the most predominant strains of reovirus belonged to GC1 in 2016, autogenous vaccines with two GC1 and one GC5 variant isolates were prepared to be used in breeders that supply chickens to the state of California. Our hypothesis relies in the fact that inactivated non-homologous vaccines provide partial protection to the field challenge not eliminating viral shedding in the infected birds; allowing the selection of strains different from GC1 and/or GC5 altering the representation of ARV’s in the environment. However, this explanation would not explain why GC4 or GC3 were not selected. We hypothesize that those genotypes were not selected due to their lack of fitness in the current environment being GC6 more fit than the rest of the genotypes. Traditionally, reported surveillance efforts do not discuss the variation of GC detections by year^2,6,20^. Since these viruses are extremely variable, their predominant genotypes change throughout time. It is important to consider GC’s predominance as a method of antigen selection for autogenous vaccine candidates.

In addition to calculating the GC frequencies temporally, we calculated homologies to a reference strain, in this case the commercial vaccine strain i.e. S1133. The advantage is to follow each of the cluster’s variant variability and assess if there are major changes throughout time. We found that GC1 had the highest homology. Even though, GC1 is the group that encompasses the vaccine strains, the average homology of this group was 77%. The rest of the GCs had average homologies to S1133 between 58.5 and 53.1%, very distant from the viruses that are used in commercial live and inactivated vaccines (S1133, 1733 and 2408). These results might be explaining the lack of effectiveness of these vaccines in protecting commercial broilers. Based on the homologies over time, we see that each of the clusters have maintained their homologies to S1133 since 2016 (Fig. 2).

The objective of performing whole genome sequencing on the seven selected isolates was to examine the variability of the different reoviruses by gene and evaluate the influence that each gene has on the whole virus variability. The oldest strain in the phylogenetic tree, i.e. an ARV from 1970 reported by Liu^9^ (T6), grouped closer to vaccine strains S1133 and 2408 in all the examined genes. Another strain from Taiwan, i.e. 750505, from 1986 did not show a defined pattern in the studied genes. Interestingly, Taiwanese strains isolated in 1992, i.e. 919, 601 G, R2/TW, 918, 916 and 1017-1, interrelate with current strains from California in M class and S class genes specifically S1 and S3. This observation suggests major genetic changes in part of the genome of ARV’s prior to 1990 and the emergence of ARV genetic variants.

Our sequencing results and the % identities of each of the viral genes with S1133, indicated that the L3, S1 and M2 genes, coding for proteins located in the virus capsid, were the genes that accounted for most of the variability of these reoviruses. The location of the proteins that they encode for, suggest a potential role in viral antigenicity and pathogenicity. In 2006, Su and collaborators described the sequence divergence of the M2 gene using the M-class genome segments of 12 distinct avian reovirus strains^25^. They deduced that the M2 gene and μB protein showed the greatest level of sequence divergence, partially confirming our results. However, no correlation with antigenicity and pathogenicity was detected. These findings should be considered in future studies in order to associate these genes variability with antigenicity and pathogenicity. Hsu and collaborators demonstrated the effectiveness of monoclonal antibodies in σC epitope recognition compared to S1133 polyclonal antibodies^26^. In the future, if a clear association between genetics and antigenicity or pathogenicity is found, sequencing and characterization of these genes might generate a tool for a better and more comprehensive characterization. In regard to variability generated by reassortment, the comparison of the phylogenetic trees for each segment show that sequences of the same isolates consistently clustered together, i.e. the four isolates CAT1600137, CAT1600260, CAK1600402 and CAK1600600 as well as the two isolates K1502030 and T1502036. This clustering pattern is an indication that no reassortment happened. The information generated in the present study helps us understand the epidemiology of reoviruses in California. In addition, provides insights on how other genes that are not commonly studied add variability to the reovirus genome.

## Methods

### Ethics statement

Tissue collections for virus isolation were conducted in accordance with procedural guidelines approved by the United States Department of Agriculture (USDA)(http://www.aphis.usda.gov/animal_health/lab_info_services/downloads/necropsyGuideline.pdf). Virus isolation and biological use was approved by the University of California, Davis Institutional Biosafety Committee (IBC) under Biological Use Authorization (BUA) approval # R2109. Procedures involving animals were reviewed and authorized by the University of California Institutional Animal Care and Use Committee (IACUC) Approval # 19092.

### ARV isolation

Tendon, heart, joint, intestine, liver and pancreas from broiler chickens suspected of tenosynovitis and pericarditis were individually minced with a scalpel and homogenized in viral transport media (VTM) using a gentleMACS Octo dissociator (Milteny Biotech, Bergisch Gladbach, Germany). Homogenized samples were diluted in VTM to a concentration of 1:10 weight/volume and syringe filtered through a 0.2-micron sterile filter. One millilitre of filtered homogenate was inoculated onto 70–90% confluent chicken embryo liver (CEL) cells in 12.5 cm^2^ tissue culture flasks and incubated at 37 °C for 1 hour. The cells were rinsed with 2 ml of Hank’s balanced salt solution and 2.5 ml of 1% fetal bovine serum (FBS) maintenance medium was added to each flask. The flasks were kept in a 5% CO_2_ incubator at 37 °C for up to 5 days. The flasks were observed daily, compared to a negative control flask, for the development of characteristic cytopathic effect (CPE). Samples that showed evidence of reovirus-like CPE were submitted for RT-PCR confirmation of a conserved segment of the S4 gene^24^. Samples with no visible CPE after 5 days were freeze/thawed 3 times and re-inoculated onto fresh CEL cells for a 2^nd^ passage.

### Reovirus molecular characterization

One hundred and fifty virus isolates obtained from broiler cases of tenosynovitis between September 2015 and June 2018 were selected for molecular characterization. The selection criteria involved clinical relevance, gross pathology and cytopathic effect in CEL. Isolates, were confirmed as positive by RT-PCR using the primers ARV_S4_P4 5′-GTGCGTGTT GGAGTTTC-3′ and ARV_S4_P5 5′-ACAAAGCCAGCCATRAT-3′ targeting a segment of 437 bp of the S4 gene^24^. Using primers P1 5′-AGTATTTGTGAGTACGATTG-3′ and P4 5′-GGCGCCACACCTTAGGT-3′ a segment of 1,088 bp of the S1 gene was amplified and studied^2^. Positive samples were sent for sequencing using forward and reverse primers to obtain the 1,088 bp segment of the S1 gene^2^ (Supplemental Table 1). Nucleotide sequences were transformed into amino acid sequences. Amino acid sequence identities to the vaccine strain (S1133) was calculated from the effectively sequenced isolates, using Clustal Omega^27^. Sequence alignments were crafted, using MEGA7^28^ including 49 reference sequences obtained from the available literature^2,6,9,11,20,22^ and three sequences representing commercially available vaccines: S1133 (AF330703), 1733 (AF004857) and 2408 (AF204945). The 49 sequences obtained from the literature represented the previously described genotypic groups for ARV (Supplemental Table 2). A segment of 278 amino acids corresponding to position 678 to 1,512 of the full S1 gene of each ARV strain was used in the phylogenetic analysis. This analysis was performed using maximum likelihood method with 1,000 bootstrap replicates using MEGA7^28^. Phylogenetic trees were generated using FigTree^29^ obtaining a visual representation of the genetic clusters.

### Whole genome sequencing of ARV isolates

In order to determine the variability of the ARV genes, seven ARV isolates were selected, based on clinical signs severity in the field, tissue of isolation (tendon/joints and heart) and CPE in cells, and submitted to full genome sequencing: K1502030, T1502036, T1600137, T1600260, K1600402, K1600600 and K1600657. Extraction of RNA was completed from 100 μl of the isolate using Trizol (ThermoFisher, Waltham, MA). Deoxyribonucleic acid (DNA) was removed using the Turbo DNA-free kit, followed by rRNA depletion using the Terminator 5′-Phosphate-Dependent Exonuclease (Epicentre Biotechnologies, Madison, WI) according to the manufacturer’s instructions. After stopping the reaction by adding Ethylenediaminetetraacetic acid (EDTA) to a concentration of 5 mMol, it was cleaned up using the QIAamp Viral RNA Mini kit (Qiagen, Valencia, CA) without the addition of carrier RNA. The elution volume was 30 μl. rRNA contamination was evaluated by RNA pico chip using a 2100 Bioanalyzer (Agilent, Santa Clara, CA). The cDNA libraries were prepared using the NEB Next Ultra Directional RNA Library Prep Kit for Illumina (New England BioLabs, Ipswich, MA). Sequencing was performed using Illumina HiSeq 3000 at the 100 bp paired end. Raw reads were aligned to the chicken genome (galGal5) using Tophat 2^30^ with default parameters. Contigs were built from the non-host reads and viral contigs were determined by using NCBI-BLAST with default parameters to find contigs with sequences matching GenBank reovirus sequences. The non-host sequences were then aligned to the identified viral contigs using Tophat 2 to determine the number of viral reads. The obtained gene sequences were compared to the vaccine strain S1133 full genome available at GenBank (KF741756 to KF741765). Sequence homologies to S1133 were calculated. The seven full genome-sequenced ARV’s, in addition to 26 chicken field ARV and 2 ARV vaccine whole genomes obtained from GenBank (Table 5), were aligned and phylogenetic trees were constructed for each of their genes using Clustal Omega (Cambridgeshire, U.K.).

**Table 5:** Avian Reovirus (ARV) GenBank accession numbers by gene of 7 ARV isolates from California plus 26 field isolate and 2 vaccine sequences (S1133 and 2408) used as backbone for phylogenetic tree analysis. Country of isolation and authors are also reported.

#	Strain nomenclature	GenBank accession number of each segment										Country	Authors
		L1	L2	L3	M1	M2	M3	S1	S2	S3	S4		
1	Strain S1133	KF741756	KF741757	KF741758	KF741759	KF741760	KF741761	KF741762	KF741763	KF741764	KF741765	China	Teng *et al*. 2013
2	SD10-1	KP288857	KP288858	KP288859	KP288860	KP288861	KP288862	KP288863	KP288864	KP288865	KP288866	China	Chu, 2014
3	526	KF741696	KF741697	KF741698	KF741699	KF741700	KF741701	KF741702	KF741703	KF741704	KF741705	China	Teng *et al*. 2013
4	AVS-B	FR694191	FR694192	FR694193	FR694194	FR694195	FR694196	FR694197	FR694198	FR694199	FR694200	USA	Bányai *et al*. 2016
5	C78	KF741716	KF741717	KF741718	KF741719	KF741720	KF741721	KF741722	KF741723	KF741724	KF741725	China	Teng *et al*. 2012
6	GuangxiR1	KC183748	KC183749	KC183750	KC183751	KC183752	KC183743	KC183744	KC183745	KC183746	KC183747	China	Teng *et al*. 2012
7	GuangxiR2	KF741726	KF741727	KF741728	KF741729	KF741730	KF741731	KF741732	KF741733	KF741734	KF741735	China	Teng *et al*. 2012
8	GX/2010/1	KJ476699	KJ476700	KJ476701	KJ476702	KJ476703	KJ476704	KJ476705	KJ476706	KJ476707	KJ476708	China	Li *et al*. 2014
9	GX110058	KF741736	KF741737	KF741738	KF741739	KF741740	KF741741	KF741742	KF741743	KF741744	KF741745	China	Teng *et al*. 2013
10	GX110116	KF741746	KF741747	KF741748	KF741749	KF741750	KF741751	KF741752	KF741753	KF741754	KF741755	China	Teng *et al*. 2013
11	HB10-1	KP288827	KP288828	KP288829	KP288830	KP288831	KP288832	KP288833	KP288834	KP288835	KP288836	China	Chu, 2014
12	K738/14	MF686695	MF686696	MF686697	MF686698	MF686699	MF686700	*	MF686701	MF686702	MF686703	Korea	Noh *et al*. 2017
13	LN09-1	KP288837	KP288838	KP288839	KP288840	KP288841	KP288842	KP288843	KP288844	KP288845	KP288846	China	Chu, 2014
14	PA/05682/12	KM877325	KM877326	KM877327	KM877328	KM877329	KM877330	KM877331	KM877332	KM877333	KM877334	USA	Tang and Lu, 2014
15	PA/15511/13	KP731611	KP731612	KP731613	KP731614	KP731615	KP731616	KP731617	KP731618	KP731619	KP731620	USA	Lu *et al*. 2015
16	PA/01224 A/14	KT428298	KT428299	KT428300	KT428301	KT428302	KT428303	KT428304	KT428305	KT428306	KT428307	USA	Lu and Tang, 2015
17	PA/01224B/14	KT428308	KT428309	KT428310	KT428311	KT428312	KT428313	KT428314	KT428315	KT428316	KT428317	USA	Lu and Tang, 2015
18	PA/27614/13	KU169288	KU169289	KU169290	KU169291	KU169292	KU169293	KU169294	KU169295	KU169296	KU169297	USA	Lu and Tang, 2015
19	SD09-1	KP288847	KP288848	KP288849	KP288850	KP288851	KP288852	KP288853	KP288854	KP288855	KP288856	China	Chu, 2014
20	2408	AY641742	*	AY652694	AY639613	AY635937	AY573907	AY436605^a^, AY438594^b^, AF204945^c^	*	AF208038	AF213468	USA	Liu *et al*. 2003
21	601SI	*	*	*	*	*	*	AY436599^a^, AY438588^b^, AF204947^c^	AF294769	AF208037	AF294773	Taiwan	Liu *et al*. 2003
22	916	AY641737	*	AY652701	*	*	*	AY436604^a^, AY438593^b^, AF297214^c^	AF294764	AY008383	AF294774	Taiwan	Liu *et al*. 2003
23	918	AY641738	*	AY652700	AY639617	AY635945	AY573911	AY436596^a^, AY436610^b^, AF297215^c^	AF294766	AF301473	AF294775	Taiwan	Liu *et al*. 2003
24	R2/TW	AY641744	*	*	*	*	*	AY436602^a^, AY438591^b^, AF297213^c^	AF294765	AF301472	AF294778	Taiwan	Liu *et al*. 2003
25	1017-1	AY641740	*	DQ238096	AY639611	AY635935	AY573905	AY436600^a^, AY438589^b^, AF297216^c^	AF294762	AF301474	AF294771	Taiwan	Liu *et al*. 2003
26	601 G	AY641736	*	AY652699	AY639614	AY635941	AY573908	AY436597^a^, AY436609^b^, AF297217^c^	AF311322	AY008384	AY008385	Taiwan	Liu *et al*. 2003
27	T6	DQ238094	*	AY652698	AY639621	AY635936	AY573915	AY436598^a^, AY438587^b^, AF204948^c^	AF294768	AF208036	AF213469	Taiwan	Liu *et al*. 2003
28	750505	DQ238093		AY652695	AY639615	AY635942	AY573909	AY395797^a^, AY436608^b^, AF204950^c^	AF294767	AF208035	AF213470	Taiwan	Liu *et al*. 2003
29	K1600600	MK416133	MK416134	MK416135	MK416136	MK416137	MK416138	MK416139	MK416140	MK416141	MK416142	USA	Current publication
30	K1600402	MK551735	MK551736	MK551737	MK551738	MK551739	MK551740	MK551741	MK551742	MK551743	MK551744	USA	Current publication
31	T1600260	MK554704	MK554705	MK554706	MK554707	MK554708	MK554709	MK554710	MK554711	MK554712	MK554713	USA	Current publication
32	T1600137	MK562467	MK562468	MK562469	MK562470	MK562471	MK562472	MK562473	MK562474	MK562475	MK562476	USA	Current publication
33	K1502030	MK583321	MK583322	MK583323	MK583324	MK583325	MK583326	MK583327	MK583328	MK583329	MK583330	USA	Current publication
34	K1600657	MK583331	MK583332	MK583333	MK583334	MK583335	MK583336	MK583337	MK583338	MK583339	MK583340	USA	Current publication
35	T1502036	MK616643	MK616644	MK616645	MK616646	MK616647	MK616648	MK616649	MK616650	MK616651	MK616652	USA	Current publication

a = P10; b = P17; c = Sigma C.

*No information.

## Supplementary information

Supplementary table 1 and 2
