# Supplementary material for: Genotypic Characterization of Emerging Avian Reovirus Genetic Variants in California
Source: Sci Rep. 2019 Jun 27;9:9351. doi: 10.1038/s41598-019-45494-4 (PMC6597705; doi:10.1038/s41598-019-45494-4)
Supplement: Supplementary file 1 — Supplementary table 1 and 2 [file 41598_2019_45494_MOESM1_ESM.docx]

**Genotypic Characterization of Emerging Avian Reovirus Genetic Variants in California**

S. Egaña-Labrin^A^, R. Hauck^B^, A. Figueroa^A^, S. Stoute^C^, H. L. Shivaprasad^D^, M. Crispo^C^, C. Corsiglia^F^, H. Zhou^G^, C. Kern^G^, B. Crossley^E^, R. A. Gallardo^AH^.

^A^ Department of Population Health and Reproduction, School of Veterinary Medicine, University of California, Davis

^B^ Auburn University Department of Pathobiology and Department of Poultry Science Auburn, AL.

University of California, Davis, California Animal Health & Food Safety Laboratory System,

^C^ Turlock Branch, ^D^ Tulare branch, and ^E^ Davis Branch

^F^ Foster Farms, Livingston, CA

^G^ Department of Animal Sciences, School of agriculture, University of California, Davis

^H^ Corresponding author. E-mail: [ragallardo@ucdavis.edu](mailto:ragallardo@ucdavis.edu)

**Supplementary Table 1.** List of GenBank accession numbers and supplementary information for the partial S1 sequences of the isolates present in the current research.

| **GenBank accession #** | **Isolation source** | **Year of isolation** | **Genotypic cluster partial S1 gene** |
| --- | --- | --- | --- |
| MK246972 | Heart | 2016 | 1 |
| MK246973 | Heart | 2016 | 1 |
| MK246975 | Tendon | 2017 | 1 |
| MK246978 | Tendon | 2017 | 1 |
| MK246979 | Intestine | 2017 | 1 |
| MK246980 | Intestine | 2017 | 1 |
| MK246981 | Heart | 2015 | 1 |
| MK246982 | Heart | 2016 | 1 |
| MK246983 | Joint swab | 2016 | 1 |
| MK246984 | Heart | 2016 | 1 |
| MK246985 | Tendon | 2018 | 1 |
| MK246986 | Tendon | 2016 | 1 |
| MK246989 | Joint swab | 2016 | 1 |
| MK246990 | Tendon | 2016 | 1 |
| MK246991 | Tendon | 2016 | 1 |
| MK246992 | Joint swab | 2016 | 1 |
| MK246993 | Tendon | 2016 | 1 |
| MK246995 | Joint swab | 2016 | 1 |
| MK246997 | Tendon | 2016 | 1 |
| MK246998 | Tendon | 2016 | 1 |
| MK247002 | Tendon | 2017 | 1 |
| MK247003 | Tendon | 2017 | 1 |
| MK247009 | Tendon | 2015 | 1 |
| MK247010 | * | 2016 | 1 |
| MK247011 | Joint swab | 2016 | 1 |
| MK247013 | Tendon | 2016 | 1 |
| MK247014 | Tendon | 2016 | 1 |
| MK247016 | Tendon | 2016 | 1 |
| MK247017 | Tendon | 2016 | 1 |
| MK247018 | Heart | 2016 | 1 |
| MK247019 | Tendon | 2016 | 1 |
| MK247022 | Tendon | 2016 | 1 |
| MK247026 | Tendon | 2017 | 1 |
| MK247027 | Tendon | 2017 | 1 |
| MK247028 | Tendon | 2017 | 1 |
| MK247029 | Tendon | 2017 | 1 |
| MK247030 | Intestine | 2017 | 1 |
| MK247031 | Tendon | 2017 | 1 |
| MK247032 | Tendon | 2017 | 1 |
| MK247033 | Tendon | 2017 | 1 |
| MK247039 | Tendon | 2017 | 1 |
| MK247046 | Tendon | 2017 | 1 |
| MK247047 | Tendon | 2017 | 1 |
| MK247053 | Tendon | 2017 | 1 |
| MK246977 | Tendon | 2017 | 2 |
| MK247007 | Tendon | 2018 | 2 |
| MK247012 | Heart | 2016 | 2 |
| MK247020 | Tendon | 2016 | 2 |
| MK247023 | Tendon | 2009 | 2 |
| MK247034 | Tendon | 2018 | 2 |
| MK247035 | Intestine | 2017 | 2 |
| MK247038 | Tendon | 2017 | 2 |
| MK247050 | Tendon | 2018 | 2 |
| MK247051 | Tendon | 2017 | 2 |
| MK247052 | Tendon | 2017 | 2 |
| MK247057 | Tendon | 2018 | 2 |
| MK246988 | Heart | 2016 | 3 |
| MK246974 | Heart | 2016 | 4 |
| MK246987 | Tendon | 2016 | 4 |
| MK246999 | Heart | 2017 | 4 |
| MK247001 | Pancreas | 2017 | 4 |
| MK247008 | Tendon | 2018 | 4 |
| MK247056 | Tendon | 2018 | 4 |
| MK247040 | Tendon | 2017 | 5 |
| MK247054 | Heart | 2017 | 5 |
| MK246976 | Tendon | 2017 | 6 |
| MK246994 | Tendon | 2018 | 6 |
| MK246996 | Tendon | 2016 | 6 |
| MK247000 | Tendon | 2018 | 6 |
| MK247004 | Tendon | 2017 | 6 |
| MK247005 | Tendon | 2017 | 6 |
| MK247006 | Tendon | 2017 | 6 |
| MK247015 | Tendon | 2018 | 6 |
| MK247021 | Tendon | 2016 | 6 |
| MK247024 | Tendon | 2017 | 6 |
| MK247025 | Tendon | 2017 | 6 |
| MK247036 | Tendon | 2017 | 6 |
| MK247037 | Tendon | 2017 | 6 |
| MK247041 | Tendon | 2017 | 6 |
| MK247042 | Tendon | 2017 | 6 |
| MK247043 | Tendon | 2017 | 6 |
| MK247044 | Tendon | 2017 | 6 |
| MK247045 | Tendon | 2017 | 6 |
| MK247048 | Tendon | 2017 | 6 |
| MK247049 | Tendon | 2017 | 6 |
| MK247055 | Tendon | 2018 | 6 |

*No information

**Supplementary Table 2.** List of GenBank accession numbers of the partial S1 sequences reference strains used for the phylogenetic tree classification.

| **GenBank Accession #** | **Country of isolation** | **Year of Isolation** | **Genotipic cluster partial S1 gene** | **Author** |
| --- | --- | --- | --- | --- |
| KR856980 | USA | 2014 | 2 | Lu et al., 2015 |
| KR856992 | USA | 2014 | 3 | Lu et al., 2015 |
| KR856994 | USA | 2014 | 4 | Lu et al., 2015 |
| KP727805 | USA | 2014 | 5 | Lu et al., 2015 |
| KP727783 | USA | 2012 | 6 | Lu et al., 2015 |
| KP727795 | USA | 2014 | 6 | Lu et al., 2015 |
| KJ803976 | USA | 2012 | 1 | Sellers, 2016 |
| KJ879692 | USA | 2012 | 1 | Sellers, 2016 |
| KJ879625 | USA | 2013 | 2 | Sellers, 2016 |
| KJ879682 | USA | 2013 | 2 | Sellers, 2016 |
| KJ879660 | USA | 2013 | 3 | Sellers, 2016 |
| KJ879667 | USA | 2013 | 3 | Sellers, 2016 |
| KJ803996 | USA | 2012 | 4 | Sellers, 2016 |
| KJ879644 | USA | 2013 | 4 | Sellers, 2016 |
| KJ803958 | USA | 2011 | 5 | Sellers, 2016 |
| KJ803959 | USA | 2012 | 5 | Sellers, 2016 |
| MG822695 | Canada | 2016 | 1 | Palomino et al., 2018 |
| MG822686 | Canada | 2014 | 1 | Palomino et al., 2018 |
| MG822669 | Canada | 2014 | 2 | Palomino et al., 2018 |
| MG822682 | Canada | 2014 | 2 | Palomino et al., 2018 |
| MG822679 | Canada | 2016 | 3 | Palomino et al., 2018 |
| MG822676 | Canada | 2016 | 3 | Palomino et al., 2018 |
| MG822678 | Canada | 2016 | 4 | Palomino et al., 2018 |
| MG822697 | Canada | 2015 | 4 | Palomino et al., 2018 |
| MG822690 | Canada | 2017 | 6 | Palomino et al., 2018 |
| MG822670 | Canada | 2017 | 5 | Palomino et al., 2018 |
| MG822703 | Canada | 2014 | 5 | Palomino et al., 2018 |
| L39002 | Canada | 1973 | 1 | Shapouri et al., 1995 |
| AF330703 | * | * | 1 | Bodelon et al., 2001 |
| AF004857 | * | 1997 | 1 | Vakharia et al.,1997 |
| AF204945 | USA | 1983 | 1 | Liu et al., 2003 |
| AF204950 | Taiwan | 1986 | 1 | Liu et al., 2003 |
| AF204947 | Taiwan | 1992 | 1 | Liu et al., 2003 |
| AF297214 | Taiwan | 1992 | 2 | Liu et al., 2003 |
| AF297215 | Taiwan | 1992 | 6 | Liu et al., 2003 |
| AF204948 | Taiwan | 1970 | 1 | Liu et al., 2003 |
| AF297213 | Taiwan | 1992 | 1 | Liu et al., 2003 |
| AF297216 | Taiwan | 1992 | 6 | Liu et al., 2003 |
| AF297217 | Taiwan | 1992 | 1 | Liu et al., 2003 |
| AF354224 | Germany | 1997 | 1 | Kant et al., 2003 |
| AF354226 | Germany | 1998 | 2 | Kant et al., 2003 |
| AF354227 | Germany | 1998 | 3 | Kant et al., 2003 |
| AF354221 | Germany | 1996 | 4 | Kant et al., 2003 |
| AF354230 | Germany | 1996 | 4 | Kant et al., 2003 |
| AF354219 | Germany | 1997 | 5 | Kant et al., 2003 |
| KX855920 | Canada | * | 2 | Ayalew et al., 2017 |
| KX855906 | Canada | * | 4 | Ayalew et al., 2017 |
| KX855918 | Canada | * | 5 | Ayalew et al., 2017 |
| KX855908 | Canada | * | 6 | Ayalew et al., 2017 |

*No information
